# Inhibition of mTOR signaling protects human glioma cells from hypoxia-induced cell death in an autophagy-independent manner

**DOI:** 10.1038/s41420-022-01195-y

**Published:** 2022-10-06

**Authors:** Iris Divé, Kevin Klann, Jonas B. Michaelis, Dennis Heinzen, Joachim P. Steinbach, Christian Münch, Michael W. Ronellenfitsch

**Affiliations:** 1 Dr. Senckenberg Institute of Neurooncology, University Hospital Frankfurt, Goethe University, Frankfurt am Main, Germany; 2 University Cancer Center Frankfurt (UCT), University Hospital Frankfurt, Goethe University, Frankfurt am Main, Germany; 3 Frankfurt Cancer Institute (FCI), University Hospital Frankfurt, Goethe University, Frankfurt am Main, Germany; 4 German Cancer Consortium (DKTK), Partner Site Frankfurt/Mainz, Frankfurt am Main, Germany; 5 Institute of Biochemistry II, Goethe University, Frankfurt am Main, Germany

**Keywords:** Cancer microenvironment, Drug development, Brain

## Abstract

Although malignant gliomas frequently show aberrant activation of the mammalian target of rapamycin (mTOR), mTOR inhibitors have performed poorly in clinical trials. Besides regulating cell growth and translation, mTOR controls the initiation of autophagy. By recycling cellular components, autophagy can mobilize energy resources, and has thus been attributed cancer-promoting effects. Here, we asked whether the activation of autophagy represents an escape mechanism to pharmacological mTOR inhibition in glioma cells, and explored co-treatment with mTOR and autophagy inhibitors as a therapeutic strategy. Mimicking conditions of the glioma microenvironment, glioma cells were exposed to nutrient starvation and hypoxia. We analyzed autophagic activity, cell growth, viability and oxygen consumption following (co-)treatment with the mTOR inhibitors torin2 or rapamycin, and autophagy inhibitors bafilomycin A1 or MRT68921. Changes in global proteome were quantified by mass spectrometry. In the context of hypoxia and starvation, autophagy was strongly induced in glioma cells and further increased by mTOR inhibition. While torin2 enhanced glioma cell survival, co-treatment with torin2 and bafilomycin A1 failed to promote cell death. Importantly, treatment with bafilomycin A1 alone also protected glioma cells from cell death. Mechanistically, both compounds significantly reduced cell growth and oxygen consumption. Quantitative proteomics analysis showed that bafilomycin A1 induced broad changes in the cellular proteome. More specifically, proteins downregulated by bafilomycin A1 were associated with the mitochondrial respiratory chain and ATP synthesis. Taken together, our results show that activation of autophagy does not account for the cytoprotective effects of mTOR inhibition in our in vitro model of the glioma microenvironment. Our proteomic findings suggest that the pharmacological inhibition of autophagy induces extensive changes in the cellular proteome that can support glioma cell survival under nutrient-deplete and hypoxic conditions. These findings provide a novel perspective on the complex role of autophagy in gliomas.

## Introduction

Despite some treatment options being currently available, the clinical management of glioblastoma (GB) remains challenging as tumor relapse occurs in the majority of cases. Hence, novel treatment approaches are crucial to improve the prognosis of patients diagnosed with GB. Due to its frequent aberrant activation in GB [1], the signaling cascade involving the epidermal growth factor receptor (EGFR) and the mammalian target of rapamycin (mTOR) was deemed a promising target. Yet, mTOR inhibitors have failed to improve overall survival (OS) of patients with newly diagnosed GB in clinical trials of unselected cohorts [2]. Investigating these findings, we previously demonstrated that mTOR inhibitors enhance cell survival in the context of nutrient deprivation and hypoxia in vitro [3–5]. This protective effect partly relies on metabolic changes, including reduced glucose and oxygen consumption, which, in sum, facilitate the economization of energy resources.

In addition to regulating cell growth and metabolism, mTOR complex 1 (mTORC1) plays an important role in the regulation of macroautophagy, henceforward referred to as autophagy. Under nutrient-rich conditions, mTORC1 phosphorylates the Unc-51-like kinase 1 (ULK1) at Ser 757, thereby inhibiting its activity [6]. When nutrients are scarce, mTORC1 dissociates from ULK1, allowing its activation by autophosphorylation. Alternatively, ULK1 activation can be regulated by the AMP activated protein kinase (AMPK), which can act either via direct phosphorylation of ULK1, or by inhibition of mTORC1 through activation of the tuberous sclerosis complex 2 (TSC2) [7]. Upon its activation, ULK1 recruits the autophagy-related protein 13 (ATG13), RB1-inducible coiled-coil protein 1 (FIP200) and the autophagy-related protein 101 (ATG101) to form the autophagy-initiating complex [8]. The most prominent function of autophagy is the elimination of cellular components, the recycling of which can provide an energy reservoir for cellular growth and metabolism.

While the activation of autophagy by mTOR inhibition is undisputed, studies investigating the co-inhibition of mTOR and autophagy as a therapeutic approach for malignant gliomas are sparse. In a preclinical model, the dual mTORC1/2 inhibitor NVP-BEZ235 radiosensitized the glioma cell line U251. Knockdown of autophagy-related protein 5 (ATG5) and beclin-1 augmented NVP-BEZ235-driven radiosensitization [9]. In xenografts of human primary glioma cells, combination of NVP-Bez235 and chloroquine resulted in tumor regression, whereas monotherapy with either compound only led to tumor growth arrest [10]. Both studies suggest the simultaneous inhibition of mTOR and autophagy as therapeutic strategy worth exploring in a clinical setting. However, these studies were not conducted under nutrient-deplete and hypoxic conditions, which both are dominating features of the glioma microenvironment.

For this project, we expanded on the pro-survival effects of mTOR inhibition in glioma cells previously reported by our group. We hypothesized that these effects rely on the activation of (protective) autophagy, and investigated co-treatment with mTOR inhibitors torin2 or rapamycin and autophagy inhibitors bafilomycin A1 or MRT68921. Mimicking the conditions of the glioma microenvironment by subjecting the cells to hypoxia and nutrient deprivation, we found that mTOR and autophagy inhibition, either alone or in combination, protect glioma cells from hypoxia-induced cell death. Analysis of mass spectrometry revealed that bafilomycin A1 was more potent in deregulating the cellular proteome than torin2. Proteins downregulated by bafilomycin A1 showed enrichment for mitochondrial clusters, including the mitochondrial respiratory chain, which may be advantageous for glioma cells in the tumor microenvironment.

## Results

### Activation of autophagy in response to starvation and hypoxia

**Fig. 1:**
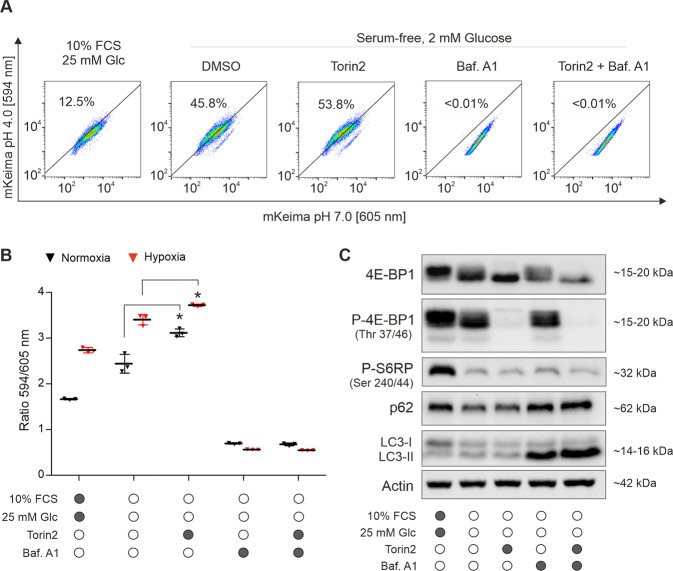
Regulation of autophagy in response to starvation, hypoxia and mTOR inhibition. **A** Autophagic activity was analyzed by flow cytometry in LN-229 cells stably expressing mKeima-LC3. Cells were cultured in hypoxia in either control or starvation medium for 24 h and were treated with torin2 (100 nM) or bafilomycin A1 (100 nM). Numbers indicate the percentage of autophagy-positive cells. **B** Quantification of flow cytometry as mean ± SD (*n* = 3, **p < 0.01, Student’s *t*-test). **C** Activation of autophagy in LN-229 cells cultured under hypoxic conditions analyzed by immunoblot. Membranes in the figure were cropped; uncropped membranes are shown in Supplementary Fig. S1. The experiment was performed twice. Abbreviations: Baf. A1= bafilomycin A1; Glc = glucose.

First, we assessed the extent of autophagy activation under the experimental conditions chosen for our in vitro model of the glioma microenvironment. To this end, we generated glioma cell lines LN-229 and LN-308 stably expressing mKeima-LC3. mKeima shows a bimodal pH-dependent excitation wavelength, shifting from 594 nm at pH 7 to 605 nm at pH 4. The ratio of 605/594 nm, assessed by flow cytometry, indicates the uptake of LC3 into the acidic lysosome and serves as marker for autophagic activity [11]. Glioma cells were exposed to glucose and serum deprivation, and cultivated either in normoxia or in hypoxia in the presence of torin2 or bafilomycin A1. Serum and glucose starvation induced an increase of the 605/594 nm ratio in LN-229 cells (Fig. 1A, B), which was further augmented by treatment with torin2. In comparison to normoxia, exposure to hypoxia was able to further enhance autophagy activation in all treatment conditions. The observed effects were abolished by co-treatment with bafilomycin A1, confirming that they were mediated by autophagy.

To confirm our findings, we performed immunoblot analyses of microtubule-associated protein 1 A/1B-light chain three (LC3) and p62, commonly used as markers for autophagy induction (Fig. 1C, Supplementary Fig. 1A–D). Serum and glucose deprivation, as well as treatment with torin2 coincided with the conversion of LC3-I to LC3-II. Simultaneously, the levels of p62 decreased, but maintained stable under (co-)treatment with bafilomycin A1. Inhibition of mTOR signaling by torin2 was confirmed by decrease in the phosphorylation of the eukaryotic translation initiation factor 4E-binding protein 1 (4E-BP1).

### Bafilomycin A1 protects glioma cells from hypoxia-induced cell death

**Fig. 2:**
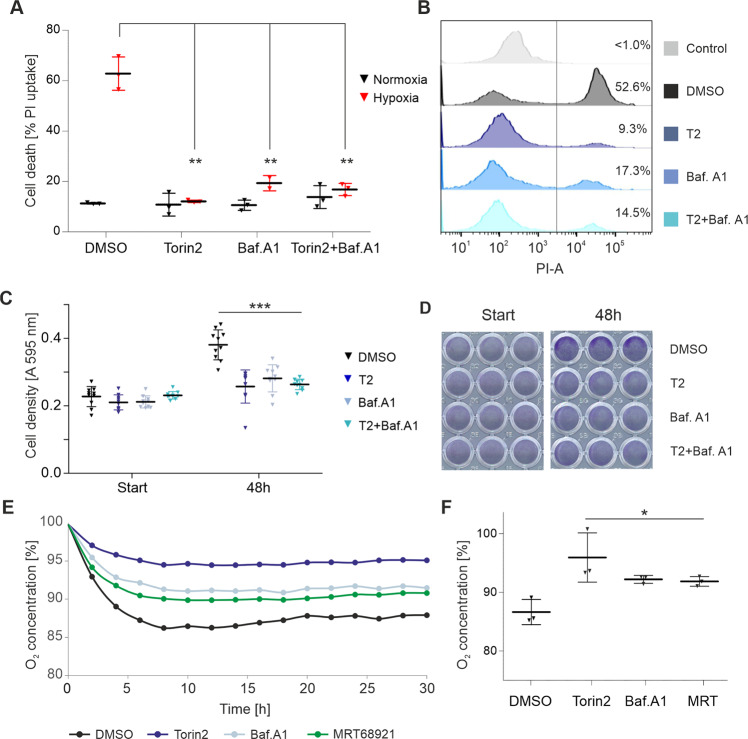
Inhibition of autophagy protects glioma cells from hypoxia-induced cell death. **A** LN-229 cells were subjected to nutrient starvation in either normoxia or 0.1% hypoxia, and were treated with torin 2 (100 nM) or bafilomycin A1 (100 nM) as indicated. Cell death was quantified by propidium iodide staining. Data represent mean ± SD (*n* = 3, **p < 0.01, Student’s *t*-test). **B** Representative histograms of **A** of cells exposed to hypoxia. PI-unstained cells served as negative control. Numbers indicate the percentage of PI-positive cells. **C**, **D** Crystal violet staining was used to quantify cell density. Data represent mean ± SD (*n* = 10, ***p < 0.001, Student’s *t*-test). **E** Quantifica*t*ion of oxygen consumption by a fluorescence-based assay. LN-229 cells were exposed to serum-free medium containing 2 mM glucose and were treated with torin 2 (100 nM), bafilomycin A1 (100 nM) or MRT68921 (1 µM) as indicated. Oxygen consumption is shown relative to the start of the experiment as mean (*n* = 3). **F** End point analysis of **E** (*n* = 3, *p < 0.05, Student’s *t*-test). Abbreviations: T2 = torin2; Baf. A1 = bafilomycin A1.

We hypothesized that mTOR inhibitors may protect glioma cells from hypoxia- and starvation-induced cell death through activation of autophagy. Hence, we investigated the effects of combined mTOR and autophagy inhibition on survival of glioma cells in the context of hypoxia and nutrient starvation. LN-229 cells were cultivated in serum-free medium containing 2 mM glucose and were exposed to 0.1% hypoxia. The cells were then treated with torin2 or bafilomycin A1 as indicated (Fig. 2A, B). Torin2 treatment led to a significant reduction of glioma cell death as shown by the percentage of PI-positive cells, thus confirming results previously reported by our group [3]. Treatment with bafilomycin A1 similarly enhanced cell survival. Most importantly, the addition of bafilomycin A1 to torin2 did not increase the percentage of PI-positive cells. Comparable results were observed in glioma cell line LN-308 (Supplementary Fig. 2A).

Using rapamycin as an alternative mTORC1-specific inhibitor in combination with bafilomycin A1 reproduced the same findings (Supplementary Fig. 2B). We also asked whether the effects observed were specific to the inhibition of late-stage autophagy, and proceeded to test MRT68921, which inhibits autophagy at early stage by blocking phosphorylation of ULK1. As observed for bafilomycin A1, MRT68921 potently protected LN-229 cells from cell death (Supplementary Fig. 2B).

### Bafilomycin A1 reduces cell density and oxygen consumption in glioma cells

We have previously reported that mTOR inhibition protects glioma cells from hypoxia-induced cell death partly through global inhibition of cellular growth and metabolism, which, in turn, helps cells to economize energy resources [4]. Based on these results, we aimed to characterize the effects of autophagy inhibition on glioma cell growth, viability and metabolism. First, we assessed cell density of LN-229 cells following treatment with bafilomycin A1 and/or torin2 (Fig. 2C, D). We found that both compounds, either alone or in combination, significantly reduced cell density after 48 h treatment duration. The reduction of cell density induced by bafilomycin A1 was dose-dependent (Supplementary Fig. 3A). Bafilomycin A1 alone did not display cytotoxicity (Supplementary Fig. 3B). The results are in line with previous reports showing that torin2 [3] and bafilomycin A1 [12] can inhibit tumor cell growth by inducing cell cycle arrest.

Next, we quantified oxygen consumption in the absence or presence of bafilomycin A1, torin2 and MRT68921. As previously published [3], torin2 profoundly reduced oxygen consumption. In addition, we found that inhibition of autophagy with both bafilomycin A1 or MRT689121 reduced oxygen consumption (Fig. 2E). The findings on cell density, cytotoxicity and oxygen consumption were confirmed in the glioma cell line LN-308 (Supplementary Fig. 4A–D).

### Changes in global proteome induced by modulation of autophagy

**Fig. 3:**
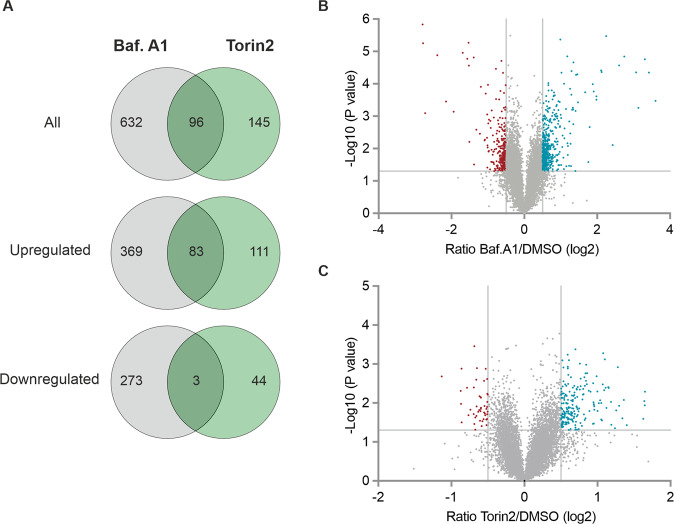
Changes in cellular proteome upon modulation of autophagy. LN-229 cells were exposed to 0.1 % hypoxia as well as serum- and glucose-starvation for 6 h and were treated with torin2 (100 nM) or bafilomycin A1 (100 nM). **A** Overlap of proteins significantly deregulated (fold change ≥ 0.5 or ≤ −0.5; *p* < 0.05) by torin2 or bafilomycin A1. **B**, **C** Volcano plot showing fold change versus *p*-value for bafilomycin A1 (B) or torin2 (C) versus DMSO.

**Fig. 4:**
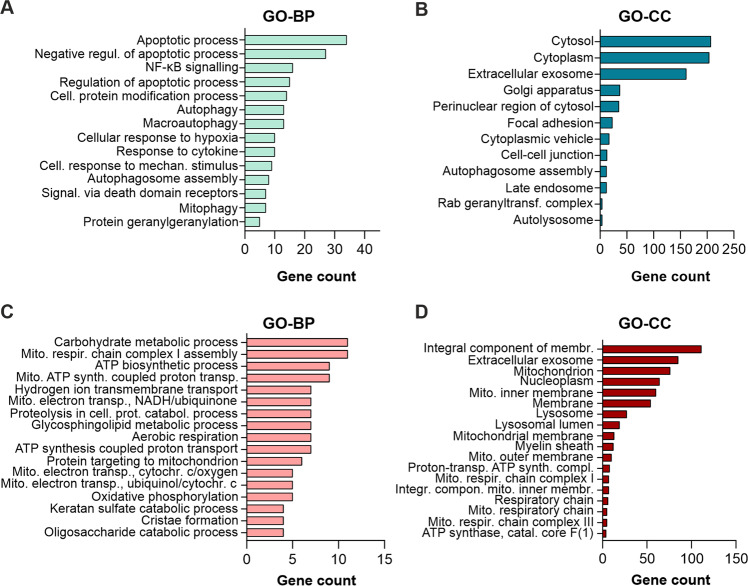
Proteomics enrichment analysis for bafilomycin A1. Bar chart showing the number of proteins upregulated (**A**, **B**) or downregulated (**C**, **D**) by bafilomycin A1 that cluster for Gene Ontology (GO) terms for Biological Process (BP) and Cellular Component (CC) with a log *p*-value of *p* < 10^−3^.

**Fig. 5:**
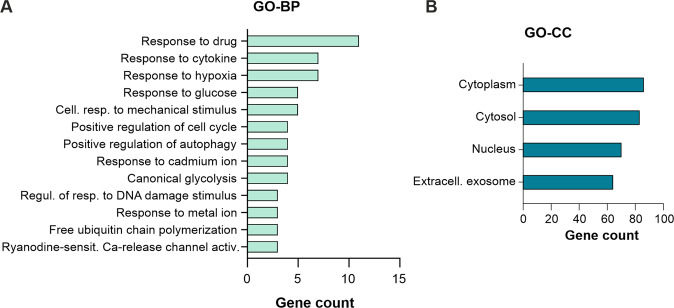
Proteomics enrichment analysis for torin2. Bar chart showing the number of proteins upregulated by torin2 that cluster for Gene Ontology (GO) terms for Biological Process (BP) (**A**) and Cellular Component (CC) (**B**) with a log *p*-value of *p* < 10^−3^.

We next aimed to study the surprising observation that activation (torin2) or inhibition (bafilomycin A1) of autophagy induced similar effects on growth and survival of glioma cells further. To identify the key networks driving these effects, we analyzed changes in the cellular proteome of LN-229 cells brought about by treatment with torin2 or bafilomycin A1 by quantitative whole-cell proteomics. We quantified proteome changes after 6 h of treatment in serum-free medium containing 2 mM glucose. Using a tandem mass tag (TMT) multiplexed-labeling approach, 7051 proteins were identified in total (supplementary Table S1). For further analysis, we chose 0.5 and −0.5 as cut-off for log_2_ fold change, and *p* < 0.05 for significance. Treatment with bafilomycin A1 significantly upregulated 452 proteins, and downregulated 276 proteins (Fig. 3A, B). In comparison, torin2 only regulated 194 and 47 proteins, respectively. Next, these proteins were subjected to the DAVID bioinformatics analysis tool for functional annotation and enrichment analysis using Gene Ontology (GO) terms Biological Process (BP) and Cellular Component (CC). Cells treated with bafilomycin A1 showed upregulation of proteins clustering within the GO-BP terms apoptotic process, response to hypoxia and cytokine, mitophagy, and (macro-)autophagy (Fig. 4A). Cytosol and cytoplasm were the predominating GO-CC terms with significantly upregulated proteins (Fig. 4B). Downregulated proteins showed enrichment for mitochondria-related GO-BP terms, such as respiratory chain complex assembly, ATP synthesis and electron transport chain, as well as oxidative phosphorylation (Fig. 4C). These clusters included subunits of the ATP synthase, the NADH/ubiquinone oxidoreductase and the cytochrome c oxidase. Accordingly, mitochondrion and mitochondrial inner membrane were among the enriched GO-CC terms with the five highest numbers of gene counts (Fig. 4D). In cells treated with torin2, upregulated proteins clustered for GO-BP terms response to drug, hypoxia and cytokine, as well as positive regulation of autophagy. Associated GO-CC terms were cytosol, cytoplasm, extracellular exosome and nucleus (Fig. 5A, B). No enriched clusters were observed for significantly downregulated proteins upon torin2 treatment, possibly due to the low number of proteins altered.

83 proteins were upregulated by both bafilomycin A1 and torin2 treatment. These included proteins associated with response to reactive oxygen species, in particular thioredoxin, glutaredoxin and Glutathione S-transferase Mu2 (GSTM2) (supplementary Table S1). Moreover, we identified proteins involved in ubiquitination (ubiquitin C-terminal hydrolase L3 (UCHL3), ubiquitin conjugating enzyme E2 L6 (UBE2L6)), regulation of apoptosis (Bcl2-associated agonist of cell death (BAD)) and transcription (Jun proto-oncogene, AP-1 transcription factor subunit (JUN)) to be upregulated by both treatments.

**Fig. 6:**
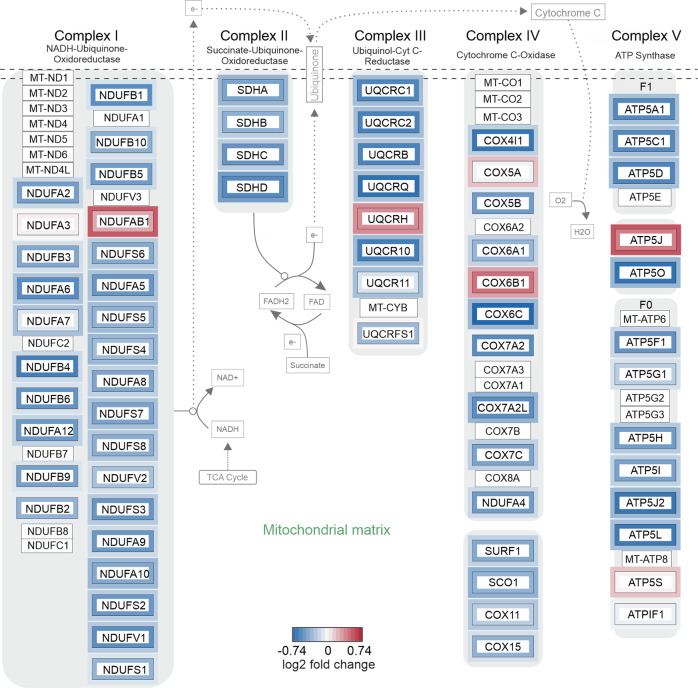
Functional enrichment analysis of proteins involved in the mitochondrial electron transport chain. Proteins were colored according to the log2 fold change induced by bafilomycin A1 (inner frame) or torin2 (outer frame).

Given the similar effects of both compounds on cellular oxygen consumption, and the striking downregulation of proteins linked to mitochondrial function in cells treated with bafilomycin A1, we screened our proteomics data for proteins involved in oxidative phosphorylation (OXPHOS). Using cytoscape and wikipathways, the log_2_ fold change of all identified proteins was subjected to pathway analysis in an unfiltered manner (Fig. 6). Exposure to bafilomycin A1 induced widespread downregulation of proteins of all mitochondrial respiratory chain complexes. For torin2, we observed a similar trend, even though fold changes were less pronounced. In sum, this analysis not only illustrates the impact of bafilomycin A1 on the global proteome, but also identifies its effects on the mitochondrial respiratory chain as explanation for the pro-survival effects observed above.

## Discussion

In this study, we investigated the role of activated autophagy as an escape mechanism to pharmacological mTOR inhibition in malignant glioma cells. As autophagy has been shown to promote tumorigenesis in a variety of tumor entities [13, 14], including gliomas [15], co-inhibition of mTOR and autophagy seems a plausible treatment approach. However, in our in vitro model of the glioma microenvironment, inhibition of early- or late-stage autophagy with MRT68921 or bafilomycin A1, respectively, increased survival of glioma cell lines LN-229 and LN-308 (Fig. 2A, B; Supplementary Fig. 1). Moreover, addition of bafilomycin A1 or MRT68921 to treatment with mTOR inhibitors rapamycin or torin2 did not enhance cell death. Thus, activation of (protective) autophagy does not account for the cytoprotective effects of pharmacological mTOR inhibition in our experimental setup. Our findings confirm that torin2 significantly activates autophagy in the cell lines used here. However, compared to serum and glucose restriction alone, the torin2-mediated additional increase of autophagic activity is significant, yet modest (Fig. 1A–C). This is in line with previous studies that identified glucose restriction as potent autophagy inducer in glioma cells [16, 17]. Based on these findings, we conclude that autophagy activation is not the driving feature of protective effects mediated by mTOR inhibition in our model, and that this may offer a plausible explanation for the failed co-treatment.

Aiming to further decipher which mechanisms drive the survival-promoting effects of torin2 and bafilomycin A1, we chose whole-cell proteomics and analyzed changes in global proteome. First, we found that bafilomycin A1 was more potent in regulating the global proteome than torin2 (Fig. 3). Similar findings were reported by a preclinical study employing an aptamer-based proteomics assay to assess protein changes in U-251 glioma cells after treatment with bafilomycin A1 or rapamycin [18]. While the superiority of bafilomycin A1 may seem surprising, given the central role of the mTOR pathway in regulating cellular protein translation [19], our immunoblot analyses show that mTOR signaling is partly suppressed by nutrient starvation and hypoxia (Fig. 1C). Though the additional blockade of mTOR by torin2 is evidenced by the decreased phosphorylation of 4E-BP1, our data imply that starvation and hypoxia are responsible for the lion’s share of proteome regulation. Interestingly, in HeLa and lung carcinoma cells A549, exposure to bafilomycin A1 or chloroquine impaired mTOR signaling as quantified by phosphorylation of S6RP [20], underlining the potential of these compounds to shape cellular pathways and metabolism. However, this stands in contrast to our results, in which the levels of P-S6RP or P-4E-BP1 were unaltered by bafilomycin A1 (Fig. 1C). This discrepancy might be due to differences in nutrient levels and treatment duration.

Second, proteins upregulated by bafilomycin A1 clustered for a variety of GO-BP terms, including negative regulation of apoptotic process, (macro-)autophagy and mitophagy, and response to hypoxia. Upregulation of autophagy-related proteins by autophagy has been described previously in mouse embryonic fibroblasts genetically lacking ATG5 or ULK1 [21]. The increase in SQSTM1/p62, optineurin (OPTN) and NBR1 autophagy cargo receptor (NBR1)—all of which were similarly elevated by bafilomycin A1 in our data—is likely to represent a compensatory mechanism to the impaired autophagic flux.

Lastly, proteins downregulated by bafilomycin A1 prominently enriched for GP-BP terms mitochondrial respiratory chain, electron transport and ATP synthesis (Fig. 4C, D). Along with this, OXPHOS pathway analysis displayed downregulation of proteins associated with complexes I to V. In line with this, bafilomycin A1, as well as MRT68921, significantly reduced cellular oxygen consumption (Fig. 2E, Supplementary Fig. 3C). Our findings are corroborated by previous studies, in which inhibition of autophagy either by genetic modification in murine skeletal muscle [22], or pharmacological inhibition by bafilomycin A1 or chloroquine in primary neurons [23] impaired mitochondrial functions and decreased the oxygen consumption rate. Mechanistically, bafilomycin A1 and chloroquine were found to impair mitochondrial quality, as assessed by mitochondrial DNA damage [23], and reduce mitochondrial network connectivity [24]. To the best of our knowledge, our study is the first to confirm the impact of bafilomycin A1 on cellular respiration in human glioma cells. Surprisingly, the torin2 data did not cluster for mitochondria-related terms, despite the well-established link of mTOR signaling to mitochondrial biogenesis and energy dynamics [25, 26]. However, in the unfiltered pathway analysis, there was a trend towards downregulation for a larger number of OXPHOS-related proteins. The fact that torin2 had a less pronounced effect on the cellular proteome, and mitochondria-related clusters in particular, may be due to the partial inhibition of the mTOR signaling cascade already by the culture conditions (low glucose and hypoxia) chosen here, as evidenced by the immunoblots (Fig. 1, Supplementary Fig. 1).

Taken together, our study shows that inhibition of mTOR protects glioma cells from hypoxia-induced cell death in an autophagy-independent manner. While the inhibition of either mTOR or autophagy differed in their potency to regulate the global proteome, similarities were found with regard to mitochondrial respiration. Our study demonstrates that modulation of autophagy can confer a survival advantage to glioma cells in the hostile conditions of the tumor microenvironment, and highlights the challenges to successfully incorporate autophagy inhibitors into glioma treatment.

## Materials and methods

### Reagents and cell lines

Reagents included torin2 and rapamycin (both Sigma-Aldrich, St. Louis, MO, USA), bafilomycin A1 (Cayman Chemicals, Ann Arbor, MI, USA) and MRT68921 (Selleck Chemicals, Houston, TX, USA). LN-229 were purchased from ATCC (Manassas, Virginia, USA). LN-308 cells were a kind gift from Prof. Nicolas de Tribolet (Lausanne, Switzerland) and were recently authenticated by short tandem repeat (STR) profiling. Cells were maintained at 37 °C and 5% CO_2_ using Dulbecco’s modified eagle medium (DMEM). The medium was supplemented with 10% fetal calf serum (Biochrom KG, Berlin, Germany), 100 µg/mL streptomycin and 100 IU/mL penicillin (Life Technologies, Karlsruhe, Germany). Cells were tested regularly for mycoplasma infection. For starvation conditions, cells were incubated in serum-free medium with 2 mM glucose. For hypoxic experimental conditions of 0.1% oxygen, we used Gas Pak pouches (Becton-Dickinson, Heidelberg, Germany).

### mKeima-LC3 assay

mKEIMA was cloned from pCHAC-mt-mKEIMA (Addgene #72342) without the mitochondrial targeting sequence into the lentiviral over-expression vector pHAGE C-TAP (gift of Prof. Wade Harper, Harvard Medical School, Boston, MA, USA). MAP1LC3B (synonym LC3B) was amplified from pDEST-GFP-LC3B (gift of Prof. Ivan Dikic, Institute of Biochemistry II, Goethe University Frankfurt, Germany) and cloned in frame C-terminal of mKEIMA with a 3x GS linker. For all cloning steps Q5® High-Fidelity DNA Polymerase (New England Biolabs, Ipswich, MA, USA, Cat#M0491) and NEBuilder® HiFi DNA Assembly (New England Biolabs, Cat#E2621) were used.

To generate lentiviral particles, HEK293T cells were transfected with pHAGE mKEIMA-LC3B and the helper vectors pHDM-VSV-G, pHDM-Hgpm2, pHDM-Tat1b and pRC-CMV-Rev1b (Addgene #164440-164443) in a ratio 20:2:1:1:1 using Lipofectamine 2000 (Thermo Fisher Scientific, Waltham, MA, USA, #11668019). Lentiviral particles were harvested in the supernatant after 24 h. Glioma cells were transduced with lentiviral particles (1:10) and transduced cells were selected with 1 μg/ml puromycin (Sigma-Aldrich) for 3 passages. Flow cytometry analysis was performed on FACSymphony A5 (BD Biosciences, Franklin Lakes, NJ, USA).

### Immunoblot

For immunoblot analysis, LN-229 cells were treated with bafilomycin A1 (100 nM) or torin2 (100 nM) for a duration of 6 h. Protein concentration was determined with the Bio-Rad Protein Assay (Bio-Rad, Munich, Germany). For SDS page analysis, 10 µg of acquired protein were used. After blocking in 5% skim milk, the membranes were incubated overnight with antibodies to S6RP (Cell Signaling, Cambridge, UK, #7217, working concentration 1:1000), P-S6RP (Ser240/44; Cell Signaling, #5364, 1:15000), 4E-BP1 (Cell Signaling, #9452, 1:1000), P-4EBP1 (Cell Signaling, #2855, 1:1000), p62 (Cell Signaling, #5114, 1:1000), Actin (Santa Cruz Biotechnology, Dallas, TX, USA, #sc-1616, 1:2000), or LC-3 (Sigma, Saint Louis, MO, USA, #L8918, 1:1000). Then, membranes were incubated for 1 h with secondary rabbit (Santa Cruz Biotechnology, #sc-2357, 1:8000) or goat antibody (Santa Cruz Biotechnology, #sc-2020, 1:3000). For detection by chemiluminescence, we used Image Lab 6.0.1 (Bio-Rad, Munich, Germany). Membranes shown in the main figures were cropped. Uncropped membranes are provided in supplementary Fig. S1.

### Cell density and viability assay

For cell growth assays, cells were seeded in 96-well plates 24 h prior to treatment. Cell density was quantified by crystal violet (CV) staining after indicated incubation times. Propidium iodide (PI) uptake was used to quantify cell death via flow cytometry as previously described [5].

### Oxygen consumption

Plates with integrated oxygen sensors (Oxo Dish OD24, PreSens, Regensburg, Germany) were used for the quantification of oxygen consumption. Cells were pre-incubated in medium containing FCS and 25 mM glucose with the indicated inhibitors for 6 h. Medium was then switched to starvation medium (serum-free, 2 mM glucose) with the indicated inhibitors. Airtight conditions were achieved by covering the wells with sterile paraffin oil. Fluorescence-based measurement of oxygen consumption was performed in triplicates.

### Mass spectrometry

For proteome analysis, cells were seeded in triplicates and were exposed to the indicated inhibitors in serum-free medium containing 2 mM glucose over a period of 6 h. The sample preparation and proteome analysis were performed as described previously [27]. For detailed information on sample preparation, fractionation and liquid chromatography mass spectrometry, please refer to supplementary file 1.

### Mass spectrometry data analysis

Raw files were analyzed using Proteome Discoverer (PD) 2.4 software (Thermo Fisher Scientific). Spectra were selected using default settings and database searches performed using Sequest HT node in PD. Database searches were performed against trypsin digested Homo Sapiens SwissProt database. Static modifications were set as TMT6 at the N-terminus and lysines and carbamidomethyl at cysteine residues. Search was performed using Sequest HT taking the following dynamic modifications into account: Oxidation (M), Met-loss (Protein N-terminus), Acetyl (Protein N-terminus) and Met-loss acetyl (Protein N-terminus). Normalized PSMs were summed for each accession and data exported for further use.

For GO-term analyses, gene sets were extracted from data using fold change and significance cutoffs as indicated. Enrichment analysis was performed with DAVID 6.8 [28] using default settings for biological processes and cellular components. For visualization of network enrichment, Cytoscape 3.9.0 software [29] was used with Omics Visualizer 1.3.0 plugin [30].

### Statistical analysis

All data is depicted as mean ± standard deviation (SD) of at least three biological replicates (*n*). Unless stated otherwise, experiments were performed three times independently. Statistical analyses were performed with Microsoft Excel 2016 (Microsoft, Redmond, WA, USA). The two-sided Student’s *t*-test was used to calculate statistical significance expressed as *p*-value. A value of *p* < 0.05 was considered to be statistically significant (*p < 0.05; **p < 0.01; ***p < 0.001). Generally, sample size calculation was conceptualized with 5% alpha error, 80% power and appropriate effect strength. Samples were only excluded from analyses due to technical problems, e.g. pipetting error, loss/spill of samples, or defects in materials/hardware. Estimate of variant was not performed prior to any statistical analyses. The variance was similar in all comparison groups.

## Supplementary information

Supplementary Figure 1

Supplementary Figure 2

Supplementary FIgure 3

Supplementary FIgure 4

Figure legends for supplementary material (clean)

Supplementary File 1

Supplementary Table 1

## Data Availability

The mass spectrometry proteomics data were deposited to the PRoteomics IDEntifications (PRIDE) Archive database of the ProteomeXchange (PX) consortium [31] with the dataset identifier PXD033503. All other datasets used in the current study are available from the corresponding author on reasonable request.
