# Supplementary figures and images for: Inhibition of mTOR signaling protects human glioma cells from hypoxia-induced cell death in an autophagy-independent manner
Source: Cell Death Discov. 2022 Oct 6;8:409. doi: 10.1038/s41420-022-01195-y (PMC9537540; doi:10.1038/s41420-022-01195-y)

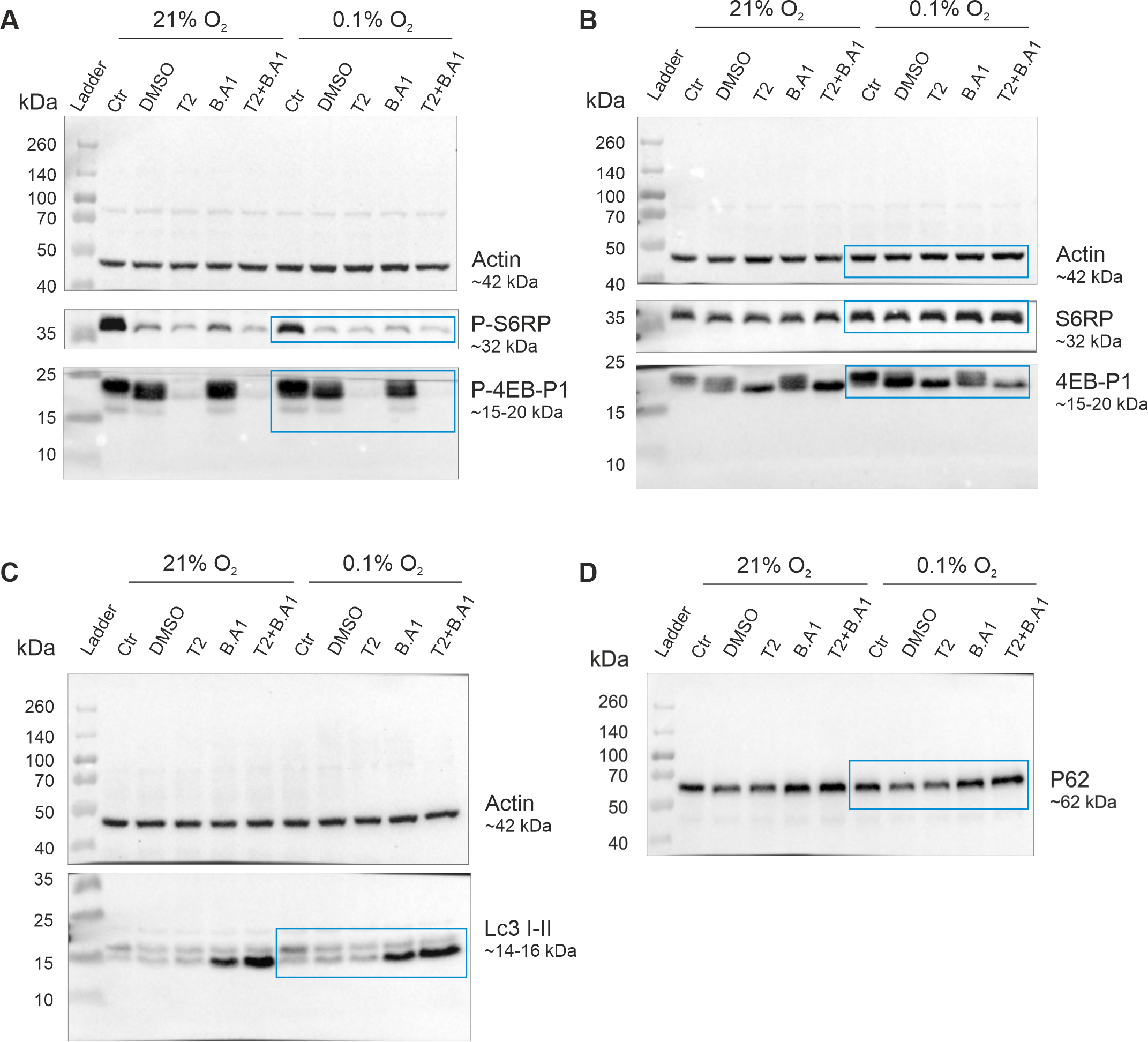

Supplement: Supplementary file 1 — Supplementary Figure 1 [file 41420_2022_1195_MOESM1_ESM.png]

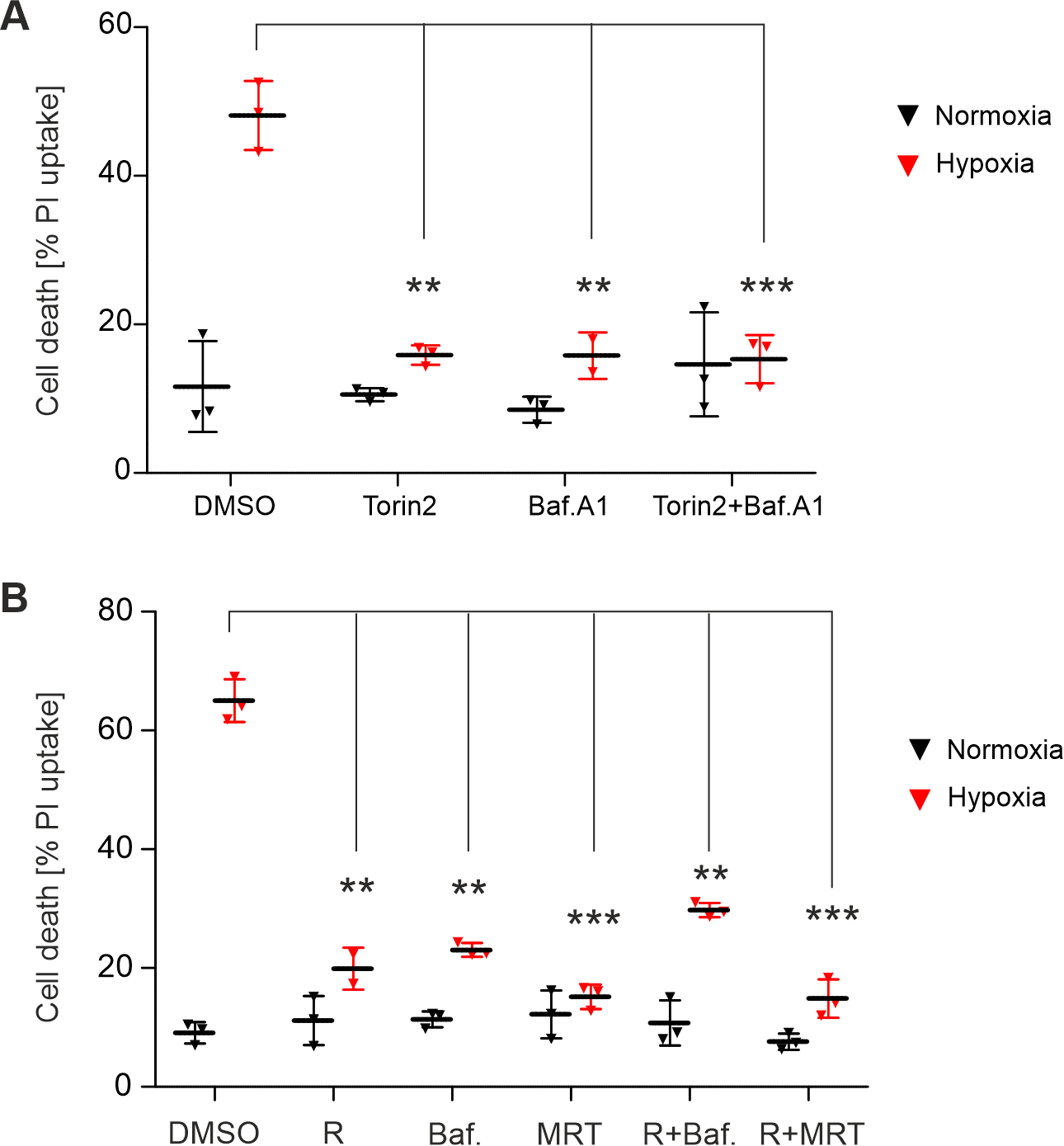

Supplement: Supplementary file 2 — Supplementary Figure 2 [file 41420_2022_1195_MOESM2_ESM.png]

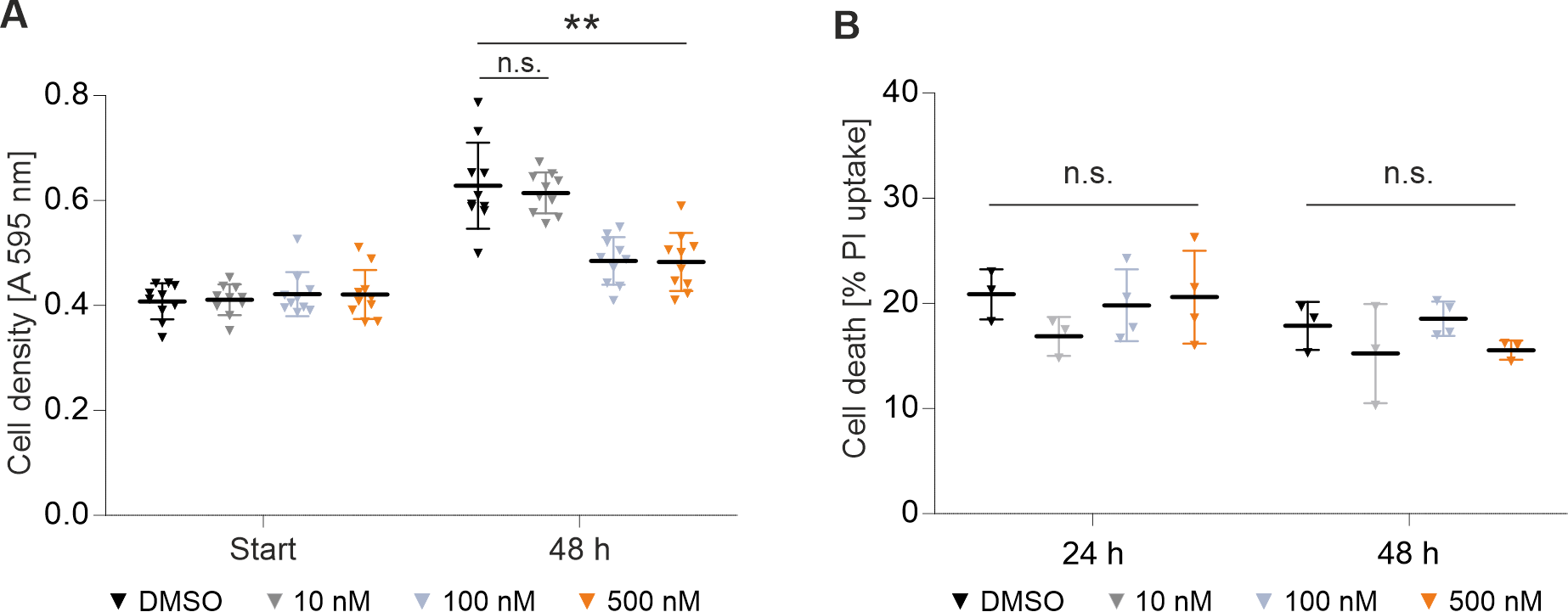

Supplement: Supplementary file 3 — Supplementary FIgure 3 [file 41420_2022_1195_MOESM3_ESM.png]

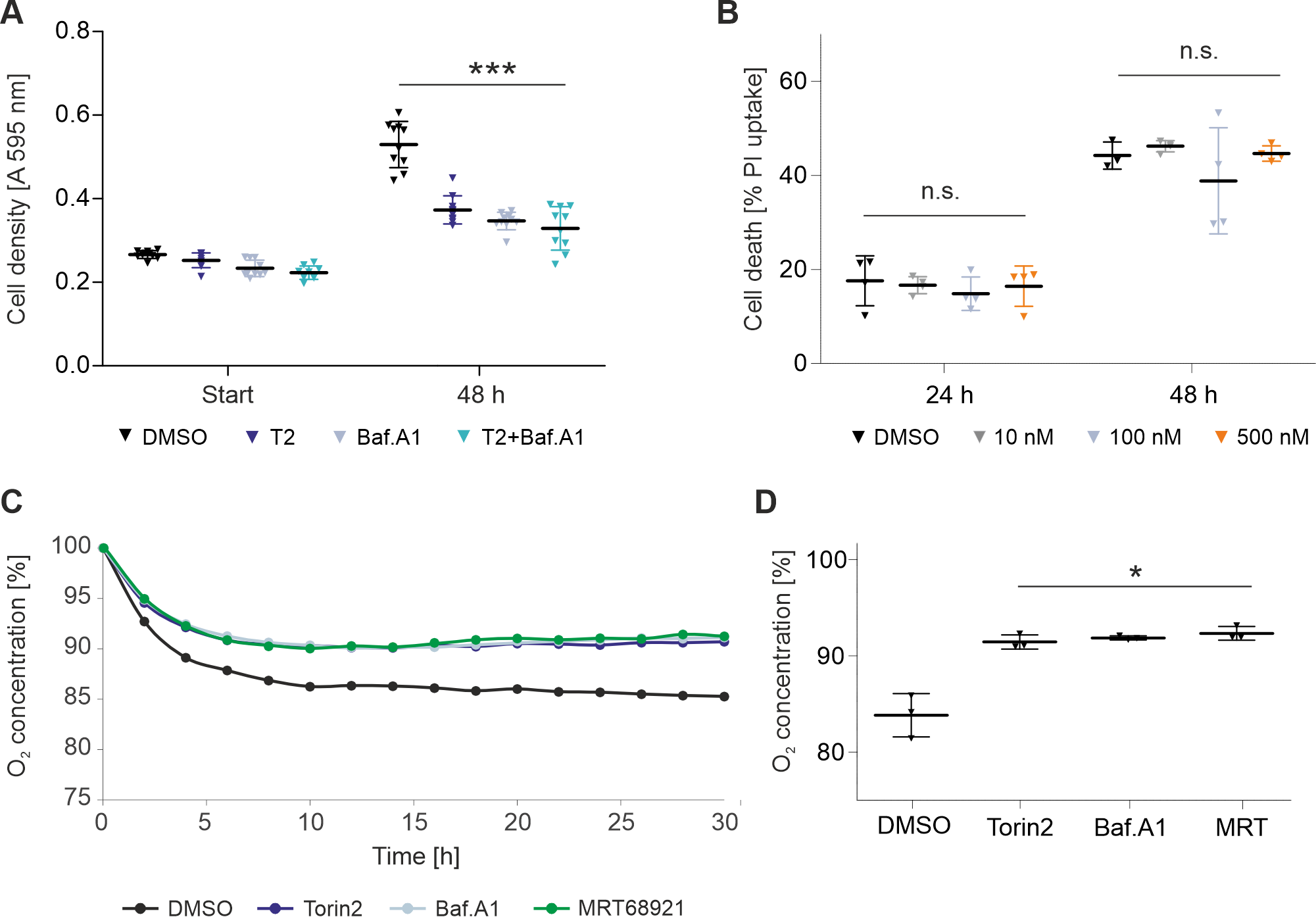

Supplement: Supplementary file 4 — Supplementary FIgure 4 [file 41420_2022_1195_MOESM4_ESM.png]
