# Supplementary material for: Inhibition of mTOR signaling protects human glioma cells from hypoxia-induced cell death in an autophagy-independent manner
Source: Cell Death Discov. 2022 Oct 6;8:409. doi: 10.1038/s41420-022-01195-y (PMC9537540; doi:10.1038/s41420-022-01195-y)
Supplement: Supplementary file 5 — Figure legends for supplementary material (clean) [file 41420_2022_1195_MOESM5_ESM.docx]

Supplementary information

Suppl. Figure 1. (**A-D**) Uncropped membranes of immunoblot analyses. LN-229 cells were cultured in serum-free medium containing 2 mM glucose and treated with bafilomycin A1 (B. A1) or torin2 (T2) in either normoxia or 0.1% hypoxia for 6 hours. DMEM containing 10% FCS and 25 mM glucose was used as control (ctr). The blue frames indicate the areas of the membranes shown in Figure 1 E.

Suppl. Fig. 2. Inhibition of autophagy protects glioma cells from hypoxia-induced cell death. (**A**) LN-308 or (**B**) LN-229 cells were subjected to nutrient starvation in either normoxia or 0.1% hypoxia, and were treated with rapamycin (100 nM), bafilomycin A1 (100 nM), torin2 (100 nM) or MRT68921 (1 µM) as indicated. Cell death was quantified by propidium iodide staining. Data represent mean ± S.D (n=3, **p<0.01, ***p<0.001, Student’s *t*-test). The experiments were performed two times. Abbreviations: Baf.=bafilomycin A1; R=rapamycin; MRT=MRT68921.

Suppl. Fig. 3. Dose-dependent impact of bafilomycin A1 on cell density and cytotoxicity. LN-229 cells were cultured in the serum-free medium containing 2 mM glucose in presence of bafilomycin A1 at indicated concentrations for a duration of 24 hours or 48 hours. (**A**) Crystal violet staining was used to quantify cell density. Data represent mean ± SD (n=10, **p<0.01, n.s. p>0.05, Student’s *t*-test). (**B**) Cell death of LN-229 cells was quantified by propidium iodide staining. n.s.=not significant. Data represent mean ± SD (n=4, n.s. p>0.05, Student’s *t*-test).

Suppl. Fig. 4. Inhibition of autophagy reduces LN-308 cell density and oxygen consumption. (**A**) Cell density was assessed by crystal violet staining following treatment with torin2 or bafilomycin A1 as indicated. Data represent mean ± SD (n=10, ***p<0.001, Student’s *t*-test). (**B**) Cytotoxicity was quantified by propidium iodide staining. Data represent mean ± SD (n=4, n.s. p>0.05, Student’s *t*-test). (**C**) LN-308 cells were cultured in serum-free medium with 2 mM glucose and treated as indicated. Oxygen consumption is shown relative to the start of the experiment as mean (n=3). (**D**) End point analysis of (C); (n=3, *p<0.05, Student’s *t*-test).
