# Supplementary material for: Inhibition of mTOR signaling protects human glioma cells from hypoxia-induced cell death in an autophagy-independent manner
Source: Cell Death Discov. 2022 Oct 6;8:409. doi: 10.1038/s41420-022-01195-y (PMC9537540; doi:10.1038/s41420-022-01195-y)
Supplement: Supplementary file 6 — Supplementary File 1 [file 41420_2022_1195_MOESM6_ESM.docx]

**Supplementary File 1:**

**Mass Spectrometry sample preparation and liquid chromatography MS**

Sample preparation for mass spectrometry

Lysates were precipitated by methanol/chloroform and proteins resuspended in 8 M Urea/10 mM EPPS pH 8.2. Concentration of proteins was determined by Bradford assay and 300 µg of protein per samples was used for digestion. For digestion, the samples were diluted to 1 M Urea with 10 mM EPPS pH 8.2 and incubated overnight with 1:50 LysC (Wako Chemicals, Neuss, Germany) and 1:100 sequencing grade trypsin (Promega, Madison, WI, USA). Digests were acidified using TFA and tryptic peptides were purified by tC18 SepPak (50 mg, Waters, Milford, MA, USA). 25 µg peptides per sample were TMT labelled and the mixing was normalized after a single injection measurement by LC-MS/MS to equimolar ratios for each channel. The pooled peptides were dried for offline High pH Reverse phase fractionation by HPLC.

Offline high pH reverse phase fractionation

Peptides were fractionated using a Dionex Ultimate 3000 analytical HPLC. 250 µg of pooled and purified TMT-labeled samples were resuspended in 10 mM ammonium-bicarbonate (ABC), 5% ACN, and separated on a 250 mm long C18 column (X-Bridge, 4.6 mm ID, 3.5 µm particle size; Waters) using a multistep gradient from 100% Solvent A (5% ACN, 10 mM ABC in water) to 60% Solvent B (90% ACN, 10 mM ABC in water) over 70 min. Eluting peptides were collected every 45 s into a total of 96 fractions, which were cross-concatenated into 12 fractions and dried for further processing.

Liquid chromatography mass spectrometry

All mass spectrometry data was acquired in centroid mode on an Orbitrap Fusion Lumos mass spectrometer hyphenated to an easy-nLC 1200 nano HPLC system using a nanoFlex ion source (ThermoFisher Scientific, Waltham, MA, USA) applying a spray voltage of 2.6 kV with the transfer tube heated to 300°C and a funnel RF of 30%. Internal mass calibration was enabled (lock mass 445.12003 m/z). Peptides were separated on a self-made, 32 cm long, 75 µm ID fused-silica column, packed in house with 1.9 µm C18 particles (ReproSil-Pur, Dr. Maisch, Ammerbuch-Entringen, Germany) and heated to 50°C using an integrated column oven (Sonation, Biberach, Germany). HPLC solvents consisted of 0.1% Formic acid in water (Buffer A) and 0.1% Formic acid, 80% acetonitrile in water (Buffer B).

For proteome analysis, a synchronous precursor selection (SPS) multi-notch MS3 method was used in order to minimize ratio compression as previously described (12). Individual peptide fractions were eluted by a non-linear gradient from 9 to 32% B over 210 minutes followed by a step-wise increase to 95% B in 16 minutes which was held for another 9 minutes. Full scan MS spectra (350-1400 m/z) were acquired with a resolution of 120 000 at m/z 200, maximum injection time of 100 ms and AGC target value of 4 x 105. The 10 most intense precursors with a charge state between 2 and 5 per full scan were selected for fragmentation (“Top 10”) and isolated with a quadrupole isolation window of 0.7 Th. MS2 scans were performed in the Ion trap (Turbo) using a maximum injection time of 85 ms, AGC target value of 2 x 104 and fragmented using CID with a normalized collision energy (NCE) of 35%. SPS-MS3 scans for quantification were performed on the 10 most intense MS2 fragment ions with an isolation window of 0.7 Th (MS) and 2 m/z (MS2). Ions were fragmented using HCD with an NCE of 65% and analyzed in the Orbitrap with a resolution of 50,000 at m/z 200, scan range of 110-500 m/z, AGC target value of 1 x105 and a maximum injection time of 86 ms. Repeated sequencing of already acquired precursors was limited by setting a dynamic exclusion of 45 seconds and 7 ppm and advanced peak determination was deactivated.
